# Preparedness of Emergency Room Nurses for Bioterrorism Based on the Health Belief Model: A Multicenter Qualitative Study

**DOI:** 10.1111/inr.70028

**Published:** 2025-05-19

**Authors:** Mi‐Kyeong Jeon, Yujeong Kim

**Affiliations:** ^1^ Department of Nursing Changwon National University Changwon Republic of Korea; ^2^ College of Nursing Research Institute of Nursing Innovation Kyungpook National University Daegu Republic of Korea

**Keywords:** bioterrorism, emergency medical services, emergency nursing, focus groups, qualitative research

## Abstract

**Aim:**

To explore emergency room nurses’ perceptions of preparedness for bioterrorism.

**Introduction:**

In the context of evolving global security threats, including wars, terrorism, and emerging infectious diseases, it is critical to evaluate the bioterrorism response competencies of emergency room nurses and identify strategies to enhance their preparedness.

**Methods:**

Qualitative data were gathered through focus group interviews with 13 emergency room nurses employed at six regional emergency centers across the country. Data were analyzed using qualitative content analysis, and findings were reported in accordance with the COREQ guidelines.

**Results:**

Analysis of 537 meaningful statements yielded 38 codes, which were organized into two themes and eight subthemes. The first theme, barriers to bioterrorism preparedness, included subthemes such as “insufficient knowledge and experience related to bioterrorism” and “contrasting thoughts on the possibility of bioterrorism occurring in South Korea.” The second theme, facilitators to bioterrorism preparedness, encompassed subthemes such as “programs to improve one's competencies in dealing with bioterrorism” and “unavoidable sense of responsibility as a nurse.”

**Conclusion:**

Despite a lack of established guidelines, knowledge, training, and response systems specific to bioterrorism, emergency room nurses demonstrate a strong sense of professional duty to provide care even in the event of a bioterrorist attack.

**Implications for Nursing and Health Policy:**

To strengthen bioterrorism preparedness, there is an urgent need to develop human resources, enhance infrastructure, and implement targeted education and training programs for healthcare professionals. Education and training are essential to enhance the competency of emergency room nurses in responding effectively to bioterrorism at all times. The inadequate bioterrorism response systems in emergency centers and the low competency levels of nurses highlight the need for policies aimed at improving bioterrorism response capabilities within the national emergency medical system.

## 1 Introduction

Bioterrorism involves the deliberate use of biological agents, such as viruses, bacteria, molds, and toxins, to inflict fatal injury or disease on humans and potentially disrupt societal structures (United Nations 2024). Global concerns regarding bioterrorism have intensified since the 2001 anthrax attacks in the United States (Ali et al. 2020). Korea, as a divided nation, faces particular vulnerabilities due to North Korea's known possession of biological weapons. Furthermore, recent conflicts, acts of terrorism, and the COVID‐19 pandemic have heightened apprehension about the potential weaponization of bioactive agents (Tin et al. 2022).

Biological agents used in bioterrorism may be naturally occurring or genetically modified microorganisms or viruses that can cause death, disease, temporary incapacitation, or permanent harm to humans, animals, and plants (United Nations 2024). The Centers for Disease Control and Prevention (CDC) classifies such agents as weapons of mass destruction, with Category A agents identified as those that are highly transmissible and associated with high mortality rates (CDC 2024). Category A agents include anthrax, botulism, plague, smallpox, tularemia, and viral hemorrhagic fevers caused by filoviruses and arenaviruses. These agents necessitate an immediate public health response due to their ease of transmission and significant lethality.

Category A agents are particularly concerning due to their ease of production, concealment, transportation, and dissemination (Broertjes et al. 2023). These agents are typically colorless, tasteless, and odorless, with latency periods that complicate the timely recognition and diagnosis of incidents (Alremeithi et al. 2023). Additionally, even small quantities of these agents can cause widespread harm, as they are capable of affecting large areas and populations. For instance, a single attack targeting public transportation or densely populated facilities could result in infections among hundreds of thousands of individuals (Zhao et al. 2023). Consequently, it is imperative that all nations establish and maintain robust response systems to counter bioterrorism threats effectively.

## 2 Background

The early detection, diagnosis, and prevention of disease spread are critical for an effective response to bioterrorism (Goniewicz et al. 2021). Given the inherent challenges in preventing bioterrorism incidents in advance, a rapid initial response following a terrorist attack is essential (Valdes and Valdes 2022). During public health crises, healthcare workers serve on the front lines, with nurses representing the largest proportion of these workers. Nurses play a pivotal role in disaster preparedness, management, emergency response, and treatment across numerous countries and constitute the majority of first responders (Said and Chiang 2020). Therefore, the awareness and response capabilities of nurses in the initial stages of a bioterrorism attack are vital for expediting diagnosis and mitigating the spread of infectious agents.

The management of patients affected by bioterrorism poses significant challenges and complexities (Rathish et al. 2022). Nurses are integral to all phases of disaster management, requiring them to rapidly identify early signs and symptoms of bioterrorism‐related infections and to isolate suspected or confirmed cases to prevent further transmission. However, nurses face an elevated risk of infection due to their proximity to patients during outbreaks, which increases the potential for community transmission (Hung et al. 2021). According to the CDC (2024), effective use of personal protective equipment (PPE), coupled with comprehensive training and education on recognizing, diagnosing, and treating infectious diseases, is critical for healthcare workers responding to bioterrorism events.

The International Council of Nurses (2019) emphasizes that early recognition of symptoms, patient isolation, and rigorous infection control measures, including equipment disinfection and linen management, are essential to mitigating transmission risks in the initial stages of a bioterrorism outbreak. Nurses must also prepare for potential occupational exposure by appropriately utilizing PPE. Beyond direct patient care, nurses play a crucial role in ensuring accurate and timely communication and addressing the psychological well‐being of isolated patients, including their anxiety and depression (Rowney and Barton 2020). Therefore, clinical nurses must be well‐versed in bioterrorism response protocols and possess the competencies to deliver first aid, provide clear communication, support community recovery, and manage mental health needs (International Council of Nurses 2019). Emergency rooms (ERs) serve as the initial treatment sites for patients following disasters. Accordingly, ER nurses must acquire and apply the requisite knowledge, skills, and competencies to deliver prompt and effective care during a bioterrorism incident (Goniewicz et al. 2021).

Lee and Kim (2021) conducted a study involving Korean nurses and identified limited awareness of bioterrorism among nurses, attributed to a lack of experience in managing patients exposed to bioterrorism. This deficiency was further linked to inadequate knowledge and coping abilities resulting from the absence of bioterrorism education within the undergraduate nursing curriculum. In contrast, the Japanese nursing curriculum mandates foundational disaster and catastrophe education during the first or second year of study, followed by a dedicated disaster nursing education course in the fourth year. This latter course emphasizes theoretical and experiential learning to equip students with the necessary knowledge, attitudes, and skills for disaster response (Han et al. 2019). Conversely, in Korea, disaster nursing education is integrated into the community nursing course, with minimal emphasis on bioterrorism, and regular bioterrorism response training is rarely conducted by medical institutions (Han et al. 2019).

Studies on nurses’ awareness of bioterrorism have revealed significant gaps. For instance, research in Poland reported that only 25% of surveyed nurses acknowledged the possibility of a bioterrorism attack (Goniewicz et al. 2021). Bioterrorism risk perception is a critical determinant of healthcare system preparedness, influencing both the willingness of healthcare workers to perform their duties under hazardous conditions and the quality of nursing services provided (Ganz et al. 2019). A study among nursing officers at Korean military hospitals also demonstrated a significant correlation between bioterrorism risk perception and preparedness (Choi and Koh 2015).

Regarding nurses’ capabilities in responding to bioterrorism, Song et al. (2021) found that Chinese nurses exhibited low levels of preparedness across prevention, preparation, and rescue dimensions. Similarly, Jiang et al. (2022) observed that Chinese intensive care unit nurses scored lowest on biological preparedness among disaster nursing competencies. In Pakistan, a study reported inadequate preparedness for large‐scale nuclear, chemical, and biological incidents among healthcare professionals, with nurses displaying lower confidence levels than doctors (Azeem et al. 2019). Research in Saudi Arabia by Nofal et al. (2021) further revealed limited bioterrorism knowledge among nurses, while a study of emergency medical personnel in Ghana found nurses to have less bioterrorism knowledge compared with other professions (Atakro et al. 2019).

A comprehensive analysis of these findings underscores the generally low levels of awareness and preparedness for bioterrorism among nurses. However, there remains a paucity of research investigating the underlying issues and strategies to address nurses’ perceptions and preparedness for bioterrorism in depth. This study aims to fill this critical gap by focusing on emergency room nurses, who play a pivotal role in the frontline response to bioterrorism incidents.

### 2.1 Aims

This study investigated ER nurses’ perceptions of preparedness against bioterrorism to provide foundational data for developing nursing education and training programs. By focusing on ER nurses, who play a critical role in frontline disaster response, it offers essential insights to improve bioterrorism preparedness within the healthcare system.

## 3 Methods

### 3.1 Design

This qualitative study employed focus group interviews (FGIs) to examine the current state of ER nurses’ preparedness for bioterrorism, as well as associated challenges, plans, and strategies. FGIs are a research method that collects data by eliciting participants’ views on a specific topic from individuals connected to that topic. Compared with one‐on‐one interviews, FGIs facilitate the generation of synergistic ideas through group interactions, making them particularly suited to exploring complex issues. Accordingly, FGIs were chosen to analyze ER nurses’ preparedness for bioterrorism. This approach allows for data collection through collective dialog, encouraging diverse perspectives and fostering dynamic discussions (Krueger and Casey 2014).

### 3.2 Theoretical Framework

The focus group questions were designed based on the health belief model (HBM) (Rosenstock et al. 1988), which explores cognitive and motivational factors influencing health behaviors. The HBM identifies key constructs that shape behavior, including perceived susceptibility (beliefs about the likelihood of experiencing a condition), perceived severity (beliefs about the seriousness of a condition and its consequences), perceived benefits (beliefs about the efficacy of actions to mitigate risks or severity), perceived barriers (beliefs about the material and psychological costs of action), cues to action (triggers that prompt behavior change), and selfefficacy (confidence in one's ability to take action). These constructs provide a robust framework for developing strategies that support both short‐ and long‐term behavior changes (Green et al. 2020) The reason for applying the HBM in this study was to analyze the factors influencing the behavioral change of ER nurses' preparedness for bioterrorism from various aspects and to explore important influencing factors by comprehensively considering the susceptibility, severity, benefits, barriers, and self‐efficacy perceived by ER nurses. Modifying factors included knowledge and prior experience related to the response to bioterrorism. In addition, perception of health risks included perceived susceptibility to the possibility of bioterrorism and perceived severity regarding the impact of bioterrorism. Likelihood of action included perceived benefits regarding whether bioterrorism response behavior would help reduce bioterrorism risks and perceived barriers regarding current problems in bioterrorism response. Cues to actions were included the influence of surroundings related to bioterrorism. Individual behaviors included self‐efficacy regarding the belief that one can respond in the bioterrorist attacks and likelihood of health behavior regarding the preparation and response strategies of ER nurses for bioterrorism.

### 3.3 Study Setting and Recruitment

The study was conducted at six regional emergency centers across South Korea. Eligibility criteria included current employment at one of these centers and a minimum of 12 months of ER experience. Participants were recruited using snowball sampling, a method where initial participants recommend others who meet the inclusion criteria. All participants received a detailed explanation of the study's objectives and methods via telephone and voluntarily consented to participate. This recruitment strategy ensured the inclusion of individuals capable of providing in‐depth and authentic insights into bioterrorism preparedness in ER settings. To ensure comprehensive data collection, a sample of at least 10 participants was targeted, consistent with qualitative research standards for capturing diverse perspectives (Freeman 2006). A total of 13 participants were interviewed, with data collection ceasing upon reaching theoretical saturation.

### 3.4 Data Collection

The primary research question guiding the in‐depth interviews was: “How prepared are ER nurses to respond to bioterrorist attacks?” Table 1 outlines the interview questions, which were developed based on the HBM through a consensus process among the investigators.

**TABLE 1:** Questions from focus group interviews guided by the health belief model.

Concept		Questions
Modifying factors		‐ How much knowledge do ER nurses have about responding to bioterrorist attacks? ‐ What experience do nurses have of education or training related to the response to bioterrorism?
Perception of health risks	Perceived susceptibility	‐ Do you think a bioterrorist attack can occur?
	Perceived severity	‐ If a bioterrorist attack occurred, how much of a threat would it be? ‐ How prepared are first responders for responding to bioterrorist attacks?
Likelihood of action	Perceived benefits	‐ Do you think that activities that improve one's competencies in responding to bioterrorist attacks can help reduce the threat from biological accidents?
	Perceived barriers	‐ What problems or obstacles are there in relation to the response to bioterrorism?
Cues to actions		‐ How have campaigns, press releases, and acquaintances affected your preparedness against bioterrorist attacks?
Individual behaviors	Self‐efficacy	‐ If a bioterrorist attack occurred, will you continue to perform nursing work? ‐ As a nurse, do you feel certain that you could respond well to bioterrorism?
	Likelihood of health behavior	‐ As an ER nurse, what do you need to prepare yourself to respond to bioterrorist attacks? ‐ What do you think are the most important factors for improving ER nurses’ preparedness against bioterrorist attack?

ER = emergency room.

Three FGIs were conducted between March 1 and June 14, 2022. Due to the COVID‐19 pandemic and the participants’ roles as ER nurses, all interviews were conducted using Zoom. According to Bender and Ewbangk (1994), a focus group typically comprises 3–12 participants. In this study, each focus group consisted of four to five nurses recruited from one or two emergency medicine centers, and a total of three FGIs were conducted. Each interview session was scheduled based on participant availability and lasted between 70 and 100 minutes. Interviews were conducted via video conferencing, with participant consent obtained beforehand. Participants were encouraged to share their thoughts, experiences, and emotions openly. The interviews continued until theoretical saturation was achieved—that is, when no new essential insights could be derived from the participants’ statements. Field notes were taken by the researcher during each FGI and were transcribed immediately afterward.

The two female researchers involved possessed substantial expertise in qualitative research. The first author has extensive experience in conducting qualitative studies and lectures on qualitative research methodology at the graduate level. The corresponding author developed an interest in bioterrorism while teaching undergraduate nursing microbiology and has significant experience participating in hospital disaster training. To ensure neutrality and minimize potential biases influencing the interviews, results, or analysis, the researchers took deliberate steps to exclude personal opinions and focused on accurately reflecting the participants’ experiences and perspectives.

### 3.5 Data Analysis

Qualitative content analysis, which is guided by research questions, was conducted using the inductive content analysis approach described by Elo and Kyngäs (2008). The process included understanding the data, open coding, grouping, categorization, and abstraction. All authors independently reviewed the interview transcriptions and field notes to develop an initial understanding of the data. Through repeated readings, they identified statements that were meaningful and relevant to the research question. Concepts and phrases that encapsulated the meaning of these statements were recorded, and each author created an independent list of open codes. These lists were then compared and integrated, with suitable names assigned to the identified meanings. Subthemes were constructed by grouping related codes, and these subthemes were further synthesized into overarching themes based on their shared meanings and characteristics.

### 3.6 Ethical Considerations

Data collection commenced following approval from the Kyungpook National University Institutional Review Board (approval number: KNU‐2022‐0005). Participant selection involved obtaining verbal consent to participate in the study, which was subsequently reconfirmed in writing immediately prior to the interviews. Before initiating the interviews, the researchers provided a comprehensive explanation of the study's objectives, the measures implemented to ensure participant privacy and anonymity, and the voluntary nature of participation, including the option to withdraw at any point. Explicit permission was obtained to record the interviews. Participants were informed that the recordings would be used solely for research purposes and would be destroyed upon the study's conclusion. Personal identifiers were removed during transcription, and unique identification numbers were assigned to safeguard participant anonymity. As a gesture of appreciation, participants were provided with gift vouchers at the conclusion of the interviews.

### 3.7 Rigor and Reflexivity

The validity of this study was ensured by adhering to Guba and Lincoln's (1989) criteria for evaluating scientific research. To establish credibility, interview recordings were transcribed verbatim, and concepts were derived by describing the content without altering the terminology used by participants. Furthermore, the interviews were conducted, and the data were analyzed with a neutral perspective to minimize researcher bias. For the criterion of fittingness, a confirmation process was conducted with two participants to verify that the researchers’ descriptions and analyses accurately reflected the participants’ experiences. Auditability was ensured through a detailed description of the analysis process provided in the Methods section. To enable verification of the findings, participant statements were directly quoted in the Findings section. Confirmability was achieved by adhering to the principles of credibility, fittingness, and auditability throughout the study.

## 4 Results

### 4.1 Characteristics of Participants

This study included 13 participants, the majority of whom were women (61.5%) and had a bachelor's degree or lower educational attainment (61.5%). The mean age of the participants was 33.46 ± 6.04 years. On average, participants had 8.79 ± 6.10 years of nursing experience, including 5.79 ± 3.29 years of experience in ER settings.

### 4.2 Themes and Subthemes

A total of 537 meaningful statements were extracted from the FGIs, which were subsequently coded into 38 categories. These categories were organized into two overarching themes and eight subthemes (Table 2).

**TABLE 2:** Themes and subthemes identified from the focus group interviews.

Themes	Subthemes	Health belief model
Barriers to bioterrorism preparedness	Insufficient knowledge and experience related to bioterrorism	Modifying factors
	Contrasting thoughts on the possibility of bioterrorism occurring in South Korea	Perceived susceptibility
	Large differences in the perceived severity of bioterrorism	Perceived severity
	Absence of specific guidelines for bioterrorism	Perceived barriers
	Lack of social awareness about bioterrorism	Cues to actions
Facilitators to bioterrorism preparedness	Programs to improve competencies in bioterrorism management	Perceived benefits
	Unavoidable sense of responsibility as a nurse	Self‐efficacy
	Improving emergency response systems for bioterrorism	Likelihood of health behavior

### 4.3 Theme 1: Barriers to Bioterrorism Preparedness

#### 4.3.1 Insufficient Knowledge and Experience Related to Bioterrorism

Participants reported limited educational exposure to bioterrorism‐related topics, primarily through coursework on public healthcare regulations, pathology, and emergency and disaster nursing. Male participants noted having received biochemical terrorism training, including gas chamber exercises, during military service, though they did not recall specific instruction on bioterrorism. Additionally, despite receiving education on disaster‐related emergency healthcare, participants did not receive specialized training or education on bioterrorism in their professional ER roles.

I haven't received any education about bioterrorism during any of my clinical training or education as an undergraduate, graduate, or at the hospital. (Participant 12)

I received this education in the army. It was biochemical training rather than bioterrorism, but it's all similar. (Participant 3)

#### 4.3.2 Contrasting Thoughts on the Possibility of Bioterrorism Occurring in South Korea

The participants expressed divergent opinions regarding the likelihood of bioterrorism occurring in South Korea. Some participants considered bioterrorism a credible threat due to the nation's geopolitical context as a divided country prone to war or terrorism. Conversely, others dismissed the possibility, citing the global enforcement of the Biological Weapons Convention as a deterrent to such attacks.

Our country is divided, the current international climate is unstable, and there is a lot of international terrorism. So, I think it is a real possibility that there could be a terrorism incident in Korea. (Participant 10)

There is a treaty that forbids the use of specific weapons, and, as I understand it, biological weapons are among the weapons you're not allowed to use. Unless it's a really extreme situation, I don't think anyone would brazenly commit an act of biological terrorism. (Participant 7)

#### 4.3.3 Large Differences in the Perceived Severity of Bioterrorism

Participants’ perceptions of the potential severity of a bioterrorist attack varied significantly. Many emphasized that the lack of awareness and preparedness would likely lead to public panic, severe consequences for the healthcare system, and potentially nationwide destabilization. However, some participants believed that the impact of bioterrorism would not be as severe as that caused by other wartime threats, such as bombings or nuclear weapons.

If a bioterrorist attack happened, given that mass deaths are the purpose, it could cause fear and panic among people and paralyze the country. (Participant 5)

Since I've not experienced bioterrorism, I think I'm more inclined to think, “It won't be me. It won't be my family.” (Participant 12)

#### 4.3.4 Absence of Specific Guidelines for Bioterrorism

Participants highlighted the challenges of delivering patient care during the COVID‐19 pandemic due to frequently changing guidelines. They expressed concern that, in the case of bioterrorism, the absence of clear and specific protocols would create significant confusion and hinder the ability to provide effective care for patients affected by bioterrorism‐related infectious diseases.

Initially, during the COVID‐19 pandemic, even though the government sent out guidelines and hospitals made their own guidelines, it was too difficult, and we were confused because the guidelines were always changing. (Participant 12)

Although there is a protocol for responding to bioterrorist attacks, it can change depending on situational variables. I think the biggest problem is that there are no guidelines for those variables. (Participant 9)

#### 4.3.5 Lack of Social Awareness About Bioterrorism

The participants identified a general lack of societal awareness and preparedness for bioterrorism. They noted insufficient interest or training among healthcare workers, including nurses, as well as a lack of public initiatives, such as evacuation drills or awareness campaigns. As a result, the participants believed that the public would fail to recognize or respond appropriately to a suspected bioterrorist event, exacerbating the spread of infectious diseases and complicating containment efforts.

In fact, there are many nurses who don't know what bioterrorism is and know nothing about its potential effects or problems. I believe a lack of interest and awareness is the biggest problem. (Participant 10)

I've never heard anything about bioterrorism training or campaigns for the general public. I've also never talked about bioterrorism with people I know. (Participant 7)

### 4.4 Theme 2: Facilitators to Bioterrorism Preparedness

#### 4.4.1 Programs to Improve Competencies in Bioterrorism Management

Participants emphasized the critical need for simulation‐based training programs to develop rapid and precise responses to bioterrorism incidents. Such training should be conducted collaboratively with governmental agencies, fire departments, law enforcement, military forces, and healthcare institutions. They further highlighted the importance of increasing alertness and awareness among healthcare workers through targeted education and training. This should include instruction on the appropriate use of PPE, effective treatment protocols, quarantine procedures for various infectious diseases, and strategies to safeguard healthcare professionals against bioterrorism‐related infectious agents.

If a bioterrorist attack were to occur, there would have to be training as part of a close‐knit cooperative system between fire departments, the police, healthcare institutions, and local government, as well as between countries. (Participant 4)

When a bioterrorism incident occurs, I think the education I received will definitely be helpful in how to evacuate, how to provide first aid, and what protective equipment to wear. And I think that if I receive education, I will be able to know the minimum awareness of bioterrorism and the minimum ways to protect myself. (Participant 6)

#### 4.4.2 Unavoidable Sense of Responsibility as a Nurse

Participants described an unwavering commitment to their professional responsibilities, even under challenging circumstances. Reflecting on their experiences during the COVID‐19 pandemic, they reported remaining on‐site to provide patient care despite adverse conditions. They expressed a similar resolve to fulfill their duties in the event of a bioterrorist attack, underscoring their professional ethos that nurses are obligated to provide care in all situations.

Of course we took this job to provide care to and treat patients. So, that's obviously our duty and what we have to do. (Participant 10)

If I am an ER nurse in a hospital, of course I have to provide care [to my patients] while protecting myself and wearing the proper protective equipment. That's not something I would worry about, even if a bioterrorist attack happened. (Participant 6)

#### 4.4.3 Improving Emergency Response Systems for Bioterrorism

Participants identified the need to strengthen emergency response systems to mitigate the spread of bioterrorism‐related infectious diseases. They recommended expanding emergency room capacity, including increasing the number of beds and quarantine facilities. Additionally, they stressed the importance of securing adequate supplies of PPE and essential medications. To prevent community transmission of infectious agents, they called for stringent management protocols for suspected cases within hospital settings.

If a bioterrorist attack happens, many patients will get injured, and ER nurses will have to provide care to trauma patients as well. I think that initial response systems to bioterrorism must be established. (Participant 1)

Usually, when these people have symptoms, they visit the ER. I think that the system that manages these patients’ data is very important. We have to isolate these people quickly and stop transmission, so if the management system is compromised, infections will spread rapidly. (Participant 11)

## 5 Discussion

In this study, we explored ER nurses’ perceptions of their preparedness for bioterrorism. Through this analysis, we identified two primary themes: “barriers to bioterrorism preparedness” and “facilitators to bioterrorism preparedness,” which were further categorized into eight subthemes.

Five subthemes emerged under the theme of “barriers to bioterrorism preparedness.” The first subtheme was identified as “insufficient knowledge and training related to bioterrorism.” According to the HBM, education serves as a critical modifying factor that can influence perceptions and behaviors. In a South Korean study, 89.0% of nurses reported that they had not received bioterrorism‐related education or training during their professional practice, and 73.9% reported a lack of such education in their undergraduate programs (Lee and Kim 2023). This highlights the pivotal role of educational experiences in enhancing bioterrorism preparedness. The Institute for National Security Strategy (2023) emphasizes the necessity of designing and delivering targeted training programs to healthcare workers to improve their readiness for bioterrorism events. Moreover, the ICN released its Core Competencies in Disaster Nursing Version 2.0 in 2019, advocating that nurses should possess knowledge about chemical, biological, radiological, and nuclear exposures and associated decontamination techniques (ICN 2019). However, the ICN competencies require further refinement to include disaster‐specific competencies and detailed educational frameworks, enabling nurses to recognize bioterrorism threats and respond effectively at each stage. Additionally, disaster‐related curricula must be enhanced in undergraduate nursing programs to equip students with foundational knowledge and skills for bioterrorism response (Xia et al. 2020).

The second subtheme was “contrasting thoughts on the possibility of bioterrorism occurring in South Korea.” Participants expressed concerns about the potential for terrorist attacks, particularly in the context of the COVID‐19 pandemic. The Institute for National Security Strategy (2023) reported heightened anxieties regarding bioterrorism during the pandemic, while the United Nations (2021) cautioned that international terrorist organizations might exploit the global disarray caused by COVID‐19 to deploy biological weapons. These concerns were compounded by increasing military tensions, including North Korean missile launches, with the United States warning of the potential for armed conflict and the necessity for South Korea to maintain constant vigilance (Yonhap News Agency 2023). On an international scale, the Biological Weapons Convention, enacted in 1975, prohibits the development, production, and stockpiling of biological and toxin weapons. As of 2024, 187 States Parties and four Signatory States have joined this convention (United Nations 2024). To mitigate the risk of bioterrorism, global adherence to the Biological Weapons Convention must be promoted, alongside strengthened international agreements and cooperation to enhance global security. Concurrently, individual nations must elevate awareness of bioterrorism risks, implement robust bioterrorism protection and response strategies, and address existing gaps through regular training and evaluation.

The third subtheme identified was “large differences in the perceived severity of bioterrorism.” Participants demonstrated substantial variation in their perceptions of bioterrorism severity. Lee and Kim (2023) reported that clinical nurses in South Korea rated the perceived severity of bioterrorism as slightly above average, with a mean score of 3.87 out of 5. Similarly, Rathish et al. (2022) emphasized the potential for biological weapons used in bioterrorism to cause widespread harm. These weapons, often dispersed as aerosols, can spread over large areas through wind, resulting in extensive injuries and a high number of fatalities, even when small quantities are released. These findings underscore the critical need for education to enhance awareness of bioterrorism among all public healthcare workers, particularly ER nurses who serve on the front lines of patient care.

The fourth subtheme was the “absence of specific guidelines for bioterrorism.” In 2001, Japan established a dedicated organization to address bioterrorism, which developed detoxification manuals for responding to biochemical terrorist attacks. These manuals are standardized and implemented by local governments under the guidance of the central government (Kim 2023). In South Korea, bioterrorism preparedness and response guidelines have been created and disseminated by the CDC. However, countries like South Korea, which lack practical experience in handling bioterrorism, would benefit from developing more comprehensive and detailed guidelines by referencing international best practices (Kim 2023). It is therefore imperative to support research in this field to create situation‐specific, actionable guidelines for healthcare workers, including ER nurses, to effectively respond to bioterrorism incidents.

The fifth subtheme was the “lack of social awareness about bioterrorism.” Building a public consensus on bioterrorism response strategies is essential and can be achieved through campaigns aimed at raising awareness among the general population. Additionally, measures to minimize exposure and prevent the transmission of infectious agents should be identified and prioritized. Comprehensive education and regular training on bioterrorism preparedness are critical not only for healthcare workers and first responders but also for the public. Park and Choi (2020) noted that disaster evacuation training is conducted biannually in response to various types of disasters; integrating bioterrorism‐specific evacuation training into existing civil defense programs would significantly improve national preparedness for such events. Furthermore, governments should prioritize bioterrorism education and outreach, conduct inspections of event sites, ensure adequate stockpiles of necessary resources, and implement additional preparedness measures, particularly for large‐scale events, to bolster readiness and response capabilities.

Three subthemes were derived from the analysis of “facilitators to bioterrorism preparedness.” The first subtheme, “programs to improve competencies in bioterrorism management,” emphasized the necessity of targeted training initiatives. Ghahremani et al. (2022) conducted a study wherein simulation training was provided to nursing students to enhance their ability to combat bioterrorism. The findings demonstrated significant improvements in participants’ bioterrorism‐related knowledge and coping performance following the training. Similarly, Tobin et al. (2020) reported that simulation training for physicians and nurses in the United States, designed to prepare them for managing the Ebola virus, led to increased confidence in bioterrorism response. At the federal level, the U.S. Federal Government and the Department of Health and Human Services have established the National Training Plan for Bioterrorism. This plan provides routine response training while also developing educational and talent programs for healthcare and field workers (White House 2022). These findings underscore the critical need for healthcare institutions to implement regular, comprehensive training programs to enhance bioterrorism preparedness. Such efforts are essential for equipping healthcare workers, including nurses, to recognize bioterrorist attacks, deliver prompt treatment, and mitigate infection spread.

The second subtheme, the “unavoidable sense of responsibility as a nurse,” reflects the inherent responsibility nurses feel in responding to bioterrorism. Nofal et al. (2021) found that 68.7% of emergency room healthcare workers expressed willingness to support their institutions in managing and controlling bioterrorist attacks. Similarly, Atakro et al. (2019) observed positive attitudes among emergency room nurses and medical officers in Ghana toward safeguarding victims of bioterrorist incidents. However, challenges remain. For example, Shin (2021) highlighted the adverse effects of long working hours and fatigue experienced by nurses during the COVID‐19 pandemic. These conditions, compounded by inadequate staffing and low wages, pose significant threats to patient safety and workforce sustainability. While nurses play a pivotal role in public healthcare, reliance on their sense of duty alone is insufficient. Policies must be implemented to provide adequate compensation, support, and safe working conditions to sustain their commitment and effectiveness.

The third subtheme, “improving emergency response systems for bioterrorism,” focuses on systemic gaps and research needs. Many bioterrorism‐related infectious diseases, such as botulism, plague, and tularemia, lack vaccines, while the protection of healthcare workers from infection remains a critical challenge. Following the 2001 anthrax attacks in the United States, significant efforts were made by various nations, including the United States, to establish bioterrorism preparedness and response programs. These programs aimed to enhance international collaboration and promote research and development in the field of bioterrorism (Nofal et al. 2021). Despite these initiatives, gaps in healthcare sector preparedness were exposed. Addressing these gaps requires urgent action by the Centers for Disease Control and Prevention (CDC) and associated agencies to develop effective vaccines and antimicrobial therapies against biological weapons (Goniewicz et al. 2021).

### 5.1 Limitations

This study has some limitations. First, the findings may not be generalizable to all nurses working in emergency medical centers as the sample included only a limited number of emergency room nurses in South Korea. Additionally, the perspectives of other healthcare professionals, such as physicians and emergency responders, may differ. Second, the participants’ statements, originally provided in Korean, were translated into English. While every effort was made to ensure accurate translation, some nuances of the native language may not have been fully captured.

### 5.2 Recommendations for Further Research

Future studies should focus on assessing the outcomes of developing and implementing diverse, situation‐specific training programs designed to equip nurses with the skills needed to respond effectively to bioterrorism. Additionally, research should include a broader range of professionals within the public healthcare sector who play critical roles during bioterrorism‐related incidents, such as physicians and emergency responders.

### 5.3 Implications for Policy and Practice

The findings of this study suggest several actionable recommendations. First, bioterrorism‐related education for nurses should begin at the undergraduate level and continue throughout their clinical practice. Professional organizations such as the ICN should categorize core competencies in disaster nursing by disaster type and establish nursing practice guidelines and educational programs tailored to each stage of a bioterrorism attack. Second, public health agencies, including the CDC, should prioritize the development of vaccines and early diagnostic methods for bioterrorism‐related diseases, as well as the creation and dissemination of educational materials to inform both healthcare professionals and the general public about the risks and appropriate responses to bioterrorism. Furthermore, medical institutions should develop detailed bioterrorism practice guidelines and conduct regular response training scenarios for healthcare providers. Third, international organizations such as the WHO and the UN should enhance global collaboration and strengthen the Biological Weapons Convention to promote international peace and security. Countries should also consider conducting bioterrorism evacuation training for the general public to minimize risks and enhance preparedness.

## 6 Conclusion

ER nurses in South Korea hold differing perspectives regarding the likelihood of bioterrorism and the associated risks it entails. Despite the lack of sufficient guidelines, knowledge, training, and response systems for managing bioterrorist incidents, nurses exhibit a strong sense of duty that would drive them to continue delivering patient care even in the event of such an attack. This study holds significance in highlighting the current state of bioterrorism preparedness, identifying existing challenges, and proposing strategies for improvement, with a focus on ER nurses who would be at the forefront of patient care during a bioterrorist event. Bioterrorism remains a tangible and pervasive global threat. The findings of this study are anticipated to inform the preparation of human resources, enhance healthcare infrastructure, and guide the development and implementation of targeted education and training programs for healthcare workers in response to bioterrorism.

## Author Contributions

Study design: MJ and YK. Data collection: MJ and YK. Data analysis: MJ and YK. Study supervision: YK. Manuscript writing: MJ and YK. Critical revisions for important intellectual content: MJ and YK.

## Ethics Statement

This study was performed in line with the principles of the Declaration of Helsinki. The study was approved by the Institutional Review Board of Kyungpook National University (no. KNU‐2022‐0005).

## Conflicts of Interest

The authors have no relevant financial or nonfinancial interests to disclose.

## Data Availability

The data that support the findings of this study are available from the corresponding author upon reasonable request.

## References

[inr70028-bib-0001] Ali, R. , I. Jan , and M. S. Malik . 2020. “Emerging Health Security Threats and Impact of Bioterrorism on the US National Security.” Global Political Review 5, no. 1: 94–103. 10.31703/gpr.2020(V-I).11.

[inr70028-bib-0002] Alremeithi, R. , N. Sullivan , H. Checkeye , M. Mazer‐Amirshahi , and A. Pourmand . 2023. “A Clinical Approach to an Unidentified Aerosolized Bioterrorism Agent: A Narrative Review for Emergency Providers.” Clinical and Experimental Emergency Medicine 10, no. 2: 147–156. 10.15441/ceem.22.412. 36796783 PMC10350351

[inr70028-bib-0003] Atakro, C. A. , S. B. Addo , J. S. Aboagye , et al. 2019. “Nurses‘ and Medical Officers’ Knowledge, Attitude, and Preparedness Toward Potential Bioterrorism Attacks.” SAGE Open Nursing 5: 2377960819844378. 10.1177/2377960819844378. 33415237 PMC7774386

[inr70028-bib-0004] Azeem, A. R. , M. W. Sharif , A. Akhtar , et al. 2019. “Perception of Preparedness of Health Care Professionals in Case of a Nuclear, Chemical, Biological Attack/Emergency in a Tertiary Care Hospital.” Cureus 11, no. 5: e4657. 10.7759/cureus.4657. 31328050 PMC6634288

[inr70028-bib-0005] Bender, D. E. , and D. Ewbank . 1994. “The Focus Group as a Tool for Health Research: Issues in Design and Analysis.” Health Transition Review 4, no. 1: 63–80. 10147164

[inr70028-bib-0006] Broertjes, J. , E. Franz , I. H. Friesema , et al. 2023. “Epidemiology of Pathogens Listed as Potential Bioterrorism Agents, the Netherlands, 2009‒2019.” Emerging Infectious Diseases 29, no. 7: 1–9. 10.3201/eid2907.221769. PMC10310391 37347519

[inr70028-bib-0007] Centers for Disease Control and Prevention . 2024. Bioterrorism Agents/Diseases (by Category), | Emergency Preparedness & Response. Available at: http://www.bt.cdc.gov/agent/agentlist‐category.asp.

[inr70028-bib-0008] Choi, J. Y. , and C. K. Koh . 2015. “The Factors Related to Bioterrorism Preparedness of Military Nursing Officers in Armed Forces Hospital.” Korean Journal of Military Nursing Research 33, no. 1: 67–82. 10.31148/kjmnr.2015.33.1.67.

[inr70028-bib-0009] Elo, S. , and H. Kyngäs . 2008. “The Qualitative Content Analysis Process.” Journal of Advanced Nursing 62, no. 1: 107–115. 10.1111/j.1365-2648.2007.04569.x. 18352969

[inr70028-bib-0010] Freeman, T. 2006. “Best Practice' in Focus Group Research: Making Sense of Different Views.” Journal of Advanced Nursing 56, no. 5: 491–497. 10.1111/j.13652648.2006.04043.x. 17078825

[inr70028-bib-0011] Ganz, F. D. , I. Margalith , J. Benbenishty , M. Hirschfeld , N. Wagner , and O. Toren . 2019. “A Conflict of Values: Nurses' Willingness to Work Under Threatening Conditions.” Journal of Nursing Scholarship 51, no. 3: 281–288. 10.1111/jnu.12466. 30775840

[inr70028-bib-0012] Ghahremani, M. , Z. Rooddehghan , S. Varaei , and S. Haghani . 2022. “Knowledge and Practice of Nursing Students Regarding Bioterrorism and Emergency Preparedness: Comparison of the Effects of Simulations and Workshop.” BMC Nursing 21, no. 1: 1–7. 10.1186/s12912-022-00917-y. 35701749 PMC9195329

[inr70028-bib-0013] Goniewicz, K. , M. Goniewicz , F. M. Burkle , and A. Khorram‐Manesh . 2021. “Cohort Research Analysis of Disaster Experience, Preparedness, and Competency‐Based Training Among Nurses.” PLoS ONE 16, no. 1: e0244488. 10.1371/journal.pone.0244488. 33417601 PMC7793243

[inr70028-bib-0014] Green, E. C. , E. M. Murphy , and K. Gryboski . 2020. “The Health Belief Model.” The Wiley Encyclopedia of Health Psychology 2: 211–214. 10.1002/9781119057840.ch68.

[inr70028-bib-0015] Guba, E. G. , and Y. S. Lincoln . 1989. Fourth Generation Evaluation. Sage.

[inr70028-bib-0016] Han, S. J. , C. M. Cho , Y. Lee , and J. Y. Chun . 2019. “Disaster Preparedness of Community Health Nurses‐Based on Disaster Prevention Assessment Tool (DPET)‐.” Crisisonomy 15: 1–12. 10.14251/crisisonomy.2019.15.5.1.

[inr70028-bib-0017] Hung, M. S. Y. , S. K. K. Lam , M. C. M. Chow , W. W. M. Ng , and O. K. Pau . 2021. “The Effectiveness of Disaster Education for Undergraduate Nursing Students' Knowledge, Willingness, and Perceived Ability: An Evaluation Study.” International Journal of Environmental Research and Public Health 18, no. 19: 10545. 10.3390/ijerph181910545. 34639845 PMC8508175

[inr70028-bib-0018] Institute for National Security Strategy . 2023. 2022 Institute for National Security Strategy Research Report: Landscape of Terrorist Threats in the Era of Post Covid‐19 and Counterterrorism Strategies for Republic of Korea. Institute for National Security Strategy, Seoul.

[inr70028-bib-0019] International Council of Nurses . 2019. Core Competencies in Disaster Nursing Version 2.0. Available at: https://www.icn.ch/system/files/2021‐07/ICN_Disaster‐Comp‐Report_WEB.pdf.

[inr70028-bib-0020] Jiang, M. , M. Sun , X. Zhang , X. R. Luan , and R. J. Li . 2022. “Disaster Nursing Competency of Intensive Care Nurses in Jinan, China: A Multicenter Cross‐Sectional Study.” Journal of Nursing Research 30, no. 3: e207. 10.1097/jnr.0000000000000492. 35446276

[inr70028-bib-0021] Kim, E. J. 2023. “Current Status and Future Challenges of Bioterrorism Response System.” Issues and Perspectives 2123: 1–4.

[inr70028-bib-0022] Krueger, R. A. , and M. A. Casey . 2014. Focus Groups: A Practical Guide for Applied Research (4th ed.). Sage.

[inr70028-bib-0023] Lee, E. , and Y. Kim . 2021. “Factors Affecting the Competency of Nursing Students Regarding Bioterrorism.” Iranian Journal of Public Health 50, no. 4: 842–843. 10.18502/ijph.v50i4.6015. 34183939 PMC8219613

[inr70028-bib-0024] Lee, S. , and Y. Kim . 2023. “Predictors of Bioterrorism Preparedness Among Clinical Nurses: A Cross‐Sectional Study.” Nurse Education Today 122: 105727. https://doi.org.libproxy.knu.ac.kr/10.1016/j.nedt.2023.105727. 36706731 10.1016/j.nedt.2023.105727

[inr70028-bib-0025] Nofal, A. , I. AlFayyad , N. AlJerian , et al. 2021. “Knowledge and Preparedness of Healthcare Providers Towards Bioterrorism.” BMC Health Services Research 21: 426. 10.1186/s12913-021-06442-z. 33952253 PMC8097244

[inr70028-bib-0026] Park, J. , and K. Choi . 2020. “A Biological Threat of North Korea and South Korea's Preparations.” Korean Journal of Military Affairs 7: 177–207. 10.33528/kjma.2020.6.7.177.

[inr70028-bib-0027] Rathish, B. , R. Pillay , A. Wilson , and V. V. Pillay . 2022. “Comprehensive Review of Bioterrorism.” In StatPearls. StatPearls Publishing. 34033376

[inr70028-bib-0028] Rosenstock, I. M. , V. J. Strecher , and M. H. Becker . 1988. “Social Learning Theory and the Health Belief Model.” Health Education & Behavior 15: 175–183. 10.1177/109019818801500203. 3378902

[inr70028-bib-0029] Rowney, R. , and G. Barton . 2020. “The Role of Public Health Nursing in Emergency Preparedness and Response.” The Nursing Clinics of North America 40, no. 3: 499–509. 10.1016/j.cnur.2005.04.005. PMC7094297 16111995

[inr70028-bib-0030] Said, N. B. , and V. C. Chiang . 2020. “The Knowledge, Skill Competencies, and Psychological Preparedness of Nurses for Disasters: A Systematic Review.” International Emergency Nursing 48: 100806. 10.1016/j.ienj.2019.100806. 31685363

[inr70028-bib-0031] Shin, K. R. 2021. “COVID‐19 Counter Measures and Action Plan for Improvement of Nursing Treatment.” Health Insurance Review & Assessment Service Research 1, no. 1: 103–107. 10.52937/hira.21.1.1.103.

[inr70028-bib-0032] Song, S. , X. Li , S. A. Bell , X. Yang , W. Zhang , et al. 2021. “Emergency Response: A Cross‐Sectional Study of Core Competencies for Nurses Regarding Major Infectious Disease Outbreaks.” Journal of Emergency Nursing 47, no. 6: 902–913. 10.1016/j.jen.2021.04.010. 34183192

[inr70028-bib-0033] Tin, D. , P. Sabeti , and G. R. Ciottone . 2022. “Bioterrorism: An Analysis of Biological Agents Used in Terrorist Events.” The American Journal of Emergency Medicine 54: 117–121. 10.1016/j.ajem.2022.01.056. 35152120 PMC8818129

[inr70028-bib-0034] Tobin, C. D. , M. Alfred , D. A. Wilson , et al. 2020. “Train‐the‐Trainer: Pilot Trial for Ebola Virus Disease Simulation Training.” Education for Health: Change in Learning & Practice 33, no. 2: 37–45. 10.4103/efh.EfH_262_19. 33318452

[inr70028-bib-0035] United Nations . 2021. Bioterrorism: Thinking the Unthinkable. Available at: https://www.un.org/en/sc/1540/documents/Chair_Statement_BioTerrorism_Livestream_Event_2021.pdf.

[inr70028-bib-0036] United Nations . 2024. About the Biological Weapons Convention: Membership and Regional Group. Available at: https://disarmament.unoda.org/biological‐weapons/about.

[inr70028-bib-0037] Valdes, J. J. , and E. R. Valdes . 2022. Biological Agents: Threat and Response. Handbook of Security Science. Springer International Publishing.

[inr70028-bib-0038] Yonhap News Agency . 2023. Chairman of the U.S. Joint Chiefs of Staff: North Korea's Nuclear Weapons and Missiles Are a Realistic Threat. The Korean Peninsula Is a Possible War Zone. Available at: https://www.yna.co.kr/view/AKR20230722023300073.

[inr70028-bib-0039] White House . 2022. National Biodefense Strategy and Implementation Plan for Countering Biological Threats, Enhancing Pandemic Preparedness, and Achieving Global Health Security. Available at: https://www.whitehouse.gov/wp‐content/uploads/2022/10/National‐Biodefense‐Strategy‐and‐Implementati/on‐Plan‐Final.pdf.

[inr70028-bib-0040] Xia, R. , S. Li , B. Chen , Q. Jin , and Z. Zhang . 2020. “Evaluating the Effectiveness of a Disaster Preparedness Nursing Education Program in Chengdu, China.” Public Health Nursing 37, no. 2: 287–294. 10.1111/phn.12685. 31709640

[inr70028-bib-0041] Zhao, F. , C. Zhao , S. Bai , L. Yao , and Y. Zhang . 2023. “Triage Algorithms for Mass‐Casualty Bioterrorism: A Systematic Review.” International Journal of Environmental Research and Public Health 20, no. 6: 5070. 10.3390/ijerph20065070. 36981980 PMC10049471

